# Evaluation of comprehensiveness and reliability of electronic health records concerning resuscitation efforts within academic intensive care units: a retrospective chart analysis

**DOI:** 10.1186/s12873-021-00462-y

**Published:** 2021-06-10

**Authors:** Michael S. Dittmar, Sabrina Zimmermann, Marcus Creutzenberg, Sylvia Bele, Diane Bitzinger, Dirk Lunz, Bernhard M. Graf, Martin Kieninger

**Affiliations:** 1 Department of Anesthesiology, Regensburg University Medical Center, Franz-Josef-Strauss-Allee 11, 93053 Regensburg, Germany; 2 Department of Forensic Psychiatry, Bezirksklinikum Regensburg, Universitätsstraße 84, 93053 Regensburg, Germany; 3 Department of Neurosurgery, Regensburg University Medical Center, Franz-Josef-Strauss-Allee 11, 93053 Regensburg, Germany

**Keywords:** Quality management, Cardiopulmonary resuscitation, Intensive care unit, Medical documentation, Patient data management systems

## Abstract

**Background:**

According to the literature, the validity and reliability of medical documentation concerning episodes of cardiopulmonary resuscitation (CPR) is suboptimal. However, little is known about documentation quality of CPR efforts during intensive care unit (ICU) stays in electronic patient data management systems (PDMS). This study analyses the reliability of CPR-related medical documentation within the ICU PDMS.

**Methods:**

In a retrospective chart analysis, PDMS records of three ICUs of a single university hospital were searched over 5 y for CPR check marks. Respective datasets were analyzed concerning data completeness and data consistency by comparing the content of three documentation forms (physicians’ log, nurses’ log, and CPR incident form), as well as physiological and therapeutic information of individual cases, for missing data and plausibility of CPR starting time and duration. To compare data reliability and completeness, a quantitative measure, the Consentaneity Index (CI), is proposed.

**Results:**

One hundred sixty-five datasets were included into the study. In 9% (*n* = 15) of cases, there was neither information on the time points of CPR initiation nor on CPR duration available in any data source. Data on CPR starting time and duration were available from at least two data sources in individual cases in 54% (*n* = 90) and 45% (*n* = 74), respectively. In these cases, the specifications of CPR starting time did differ by a median ± interquartile range of 10.0 ± 18.5 min, CPR duration by 5.0 ± 17.3 min. The CI as a marker of data reliability revealed a low consistency of CPR documentation in most cases, with more favorable results, if the time interval between the CPR episode and the time of documentation was short.

**Conclusions:**

This study reveals relevant proportions of missing and inconsistent data in electronic CPR documentation in the ICU setting. The CI is suggested as a tool for documentation quality analysis and monitoring of improvements.

## Introduction

Recording reliable and valid data on resuscitation efforts during in-hospital cardiac arrest serves the interests of patients as well as legal, quality assurance, and scientific purposes. In practice, discrepant and missing data in resuscitation records are a frequent phenomenon [1–9]. Since in operating rooms as well as in intensive care settings, automated collection of patient vital parameters, supplemented by semi-automated or manual documentation of additional details, in electronic patient data management systems (PDMS) is on the rise, the electronic record may be the only place where data on episodes of cardiopulmonary resuscitation (CPR) are kept. Experimental findings suggest, that electronic documentation might have some benefits compared to standard paper files during CPR [10–13]. However, the quality of electronic patient records concerning the documentation of CPR in intensive care units (ICU) is largely unknown.

In this retrospective study, we assessed the comprehensiveness and reliability of CPR documentation in the PDMS of three ICUs of a single academic center. For this purpose, we analyzed whether the time point and duration of resuscitation measures, the cardiac rhythm, and cardiac defibrillations were represented plausibly and consistently within the PDMS. Further, a metric for systematically comparing the consistency of time interval related CPR documentation is suggested.

## Methods

### Ethics approval and consent to participate

According to the local clinical ethics committee, no ethics approval or patient consent was necessary (inquiry no. 17–468-104, University of Regensburg Ethics Committee). The data originated from three distinct ICUs of a university hospital. No experiments on humans or animals were conducted for this study.

### Data collection

The participating ICUs use the PDMS MetaVision Suite (Fa. iMDSoft Ltd., Tel Aviv, Israel). Vital signs are recorded at an interval of 1 min. Part of the PDMS documentation is the daily measurement of the condensed, 10-item Therapeutic Intervention Scoring System (TISS), the Core-TISS-10. The Core-TISS-10 encompasses the 10 most laborious procedures from the TISS-28, with the intention to reflect the workload of critical care for the individual patient and is a component of the German reimbursement system [14, 15]. In all electronic patient records from 2012 until 2016, the electronic documentation of the Core-TISS-10 was searched for the keyword “resuscitation”.

Whenever the TISS search revealed the keyword “resuscitation”, the different documentary forms were manually searched for hints concerning details on the appointed incident. These three forms comprised of the physicians’ free text log, the nurses’ free text log, and the CPR incident documentation form. From each, the documented time point of CPR initiation and its duration were recorded for analysis. In addition, a fourth data source for determining CPR initiation and duration was analyzed, namely the recorded vital parameters and therapy details. These were manually reviewed for pre-defined indicators of CPR initiation and cessation (Table 1), respectively. The searched interval thereby reached from 15 min before the earliest mentioning of CPR initiation in the three mentioned documentation forms until 15 min after the latest mentioning of CPR termination. The interval between the earliest indicator of CPR initiation according to Table 1 until the earliest indicator of CPR cessation was considered the estimated CPR episode according to the vital signs and therapy documentation.

**Table 1:** Indicators for initiation and cessation of CPR serving as basis for estimating the CPR interval according to vital signs and therapy documentation

Indicators for initiation of CPR	Indicators for termination of CPR
Loss of SpO_2_ signal Cardiac defibrillation i.v. epinephrine 1 mg i.v. amiodarone 300 mg HR < 30 /min HR > 200 /min Documented cardiac rhythm = VF, VT, ASY, or PEA Sudden increase in F_i_O_2_ to 1.0	Death Initiation of an ECMO therapy Documented rhythm = SR, AF, or AFlut Reduction of F_i_O_2_ from 1.0 to lesser values Termination of lung ventilation

*SpO*_*2*_ peripheral oxygen saturation, *HR* heart rate, *VF* ventricular defibrillation, *VT* ventricular tachycardia, *ASY* asystoly, *PEA* pulseless electrical activity, *F*_*i*_*O*_*2*_ fraction of inspired oxygen, *ECMO* extracorporeal membrane oxygenation, *SR* sinus rhythm, *AF* atrial fibrillation, *AFlut* atrial flutter

From these data, up to four separate CPR intervals could be found in the previously mentioned four data sources documentation (physicians’ log, nurses’ log, the CPR incident form, the vital signs and therapy documentation), which were then compared for consistency. In addition, the main cardiac rhythm during CPR as well as applied electrical defibrillations were assessed.

### Exclusion criteria

Cases were excluded, if CPR was performed fully or in part outside the ICU, e.g., when patients were admitted during CPR or if CPR was necessary during patient transport outside the ward. Further, cases without manual chest compression were not included into the analysis. This was the case, whenever resuscitation efforts were described as “pharmacologic resuscitation”. Finally, all cases with a documented CPR duration greater than 120 min were removed from data consistency evaluation, because the plausibility of such events was deemed questionable.

### Consentaneity index for assessing CPR documentation quality

To assess the plausibility and consistency of the documented CPR time courses between the three forms and the vital / therapy data, we compared the entries of the various documentation forms of individual patients. Concerning the timely parallelism of the documentation, a newly defined quality indicator – the Consentaneity Index (CI) – was calculated as follows: The period of time between the earliest mentioned CPR beginning until the latest mentioned CPR ending was divided into one-minute intervals. For each interval, we assessed, in how many of the four data sources the ongoing CPR was indicated to be ongoing. The Gaussian partial sum of the resulting count for each interval was summed up. I.e., if CPR was mentioned in one form, one point was assigned. For the second mentioning within the same one-minute interval, two additional points were counted; for the third mentioning three additional points etc. Subsequently, the points of all intervals were added up, and the summed score was divided by the number of intervals and the partial sum of the number of all forms analyzed (*n*_*all*_ = 4, see Table 2a). Thus, the CI takes values between 0 (no overlap of documented CPR intervals at all) and 1 (perfect fit of CPR documentation in all data sources).

**Table 2:** Calculation of the Consentaneity Index (CI) (i = number of CI intervals examined; n = number of simultaneously appearing CPR documentation within the individual CI interval; *n*_*all*_ = number of forms examined within the study (i.e., 4); *n*_*data*_ = number of forms with CPR data; ∆ = operator for the Gaussian partial sum with ∆x = x(x + 1)/2)

a) Standard CI:	
$$ CI=\left(\sum \limits_1^i\varDelta n\right){i}^{-1}{\left(\varDelta {n}_{all}\right)}^{-1} $$	
b) Soft CI:	
$$ {CI}_{soft}=\left(\sum \limits_1^i\varDelta n\right){i}^{-1}{\left(\varDelta {n}_{data}\right)}^{-1} $$	

The CI as described above is influenced by both the consistency and the completeness of CPR documentation. Thus, data sources lacking information on a CPR event reduce the value of the CI. To allow a focus on the consistency of CPR documentation alone, the standard CI was further differentiated into a so-called soft CI, which is not influenced by empty data sources. The soft CI is calculated by substituting the number of all forms by the number of forms presenting data on the actual CPR incident (*n*_*data*_, Table 2b).

### Calculation example

*Case Vignette.*

A 60-year-old intensive care unit patient falls into ventricular fibrillation at 12:01 o’clock. Manual chest compressions are initiated immediately, inspiratory oxygen is increased to 100%, and the defibrillator is brought into action. After a single shock, sinus rhythm is restored at 12:02, and ROSC with sufficient circulation parameters is obtained.

In the physician’s log, resuscitation efforts starting at 12:01 and lasting for two minutes are documented. The nurses’ log speaks of a 3-min resuscitation interval with a starting time at 12:00. An entry is created within the CPR incident form with a single time point at 12:05. Finally, from the vital signs and therapy documentation, the first visible indicator of CPR activity is the defibrillation, which is documented at 12:02. At 12:03, inspiratory oxygen fraction is reduced to baseline settings, indicating the successful termination of CPR.

#### Calculating the standard consentaneity index

**Fig. 1:**
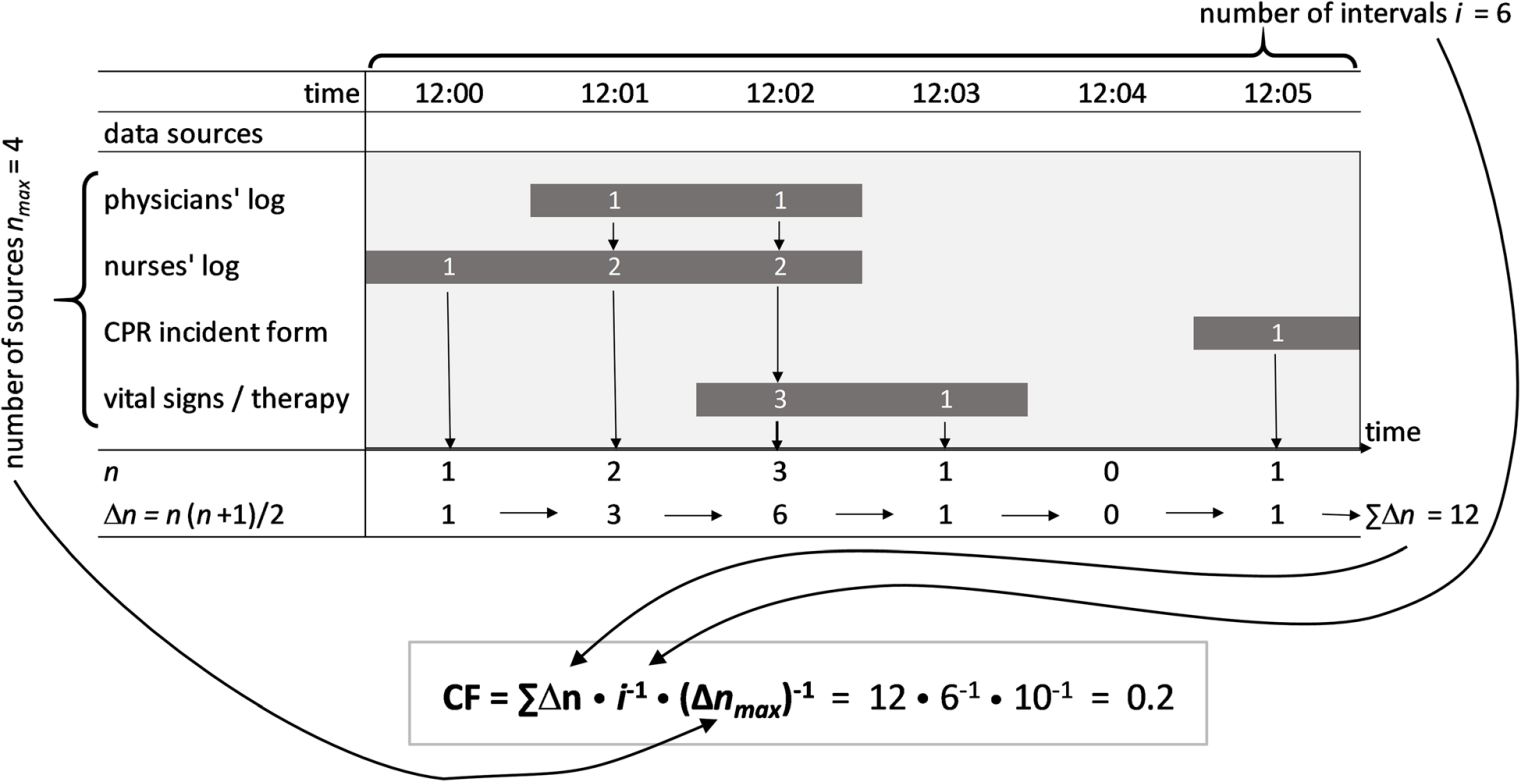
Calculation example of the standard CI

According to the clinical documentation, the CI is calculated as presented in Fig. 1. The evaluated CPR interval ranges from 12:00 to 12:05 and thus comprises 6 one-minute intervals (*i* = 6). For each minute with a single mentioning of CPR activity in the data sources (*n* = 1), one point is assigned. In case of two overlapping CPR entries (*n* = 2), three points are added (Δ2 = 3). At 12:02, three data sources report the ongoing CPR (*n* = 3), thus Δ3 = 6 points are added. Since for 12:04 there is no documentation of resuscitation efforts, for this minute no points are assigned. The sum ∑Δ*n* = 12 is subsequently divided by the number of intervals *i* = 6 and the Gaussian partial sum of the number of data sources evaluated (Δ*n*_*max*_ = 10). The calculation results in a standard CI of 0.2 (Fig. 1).

### Interpretation of the CI

**Fig. 2:**
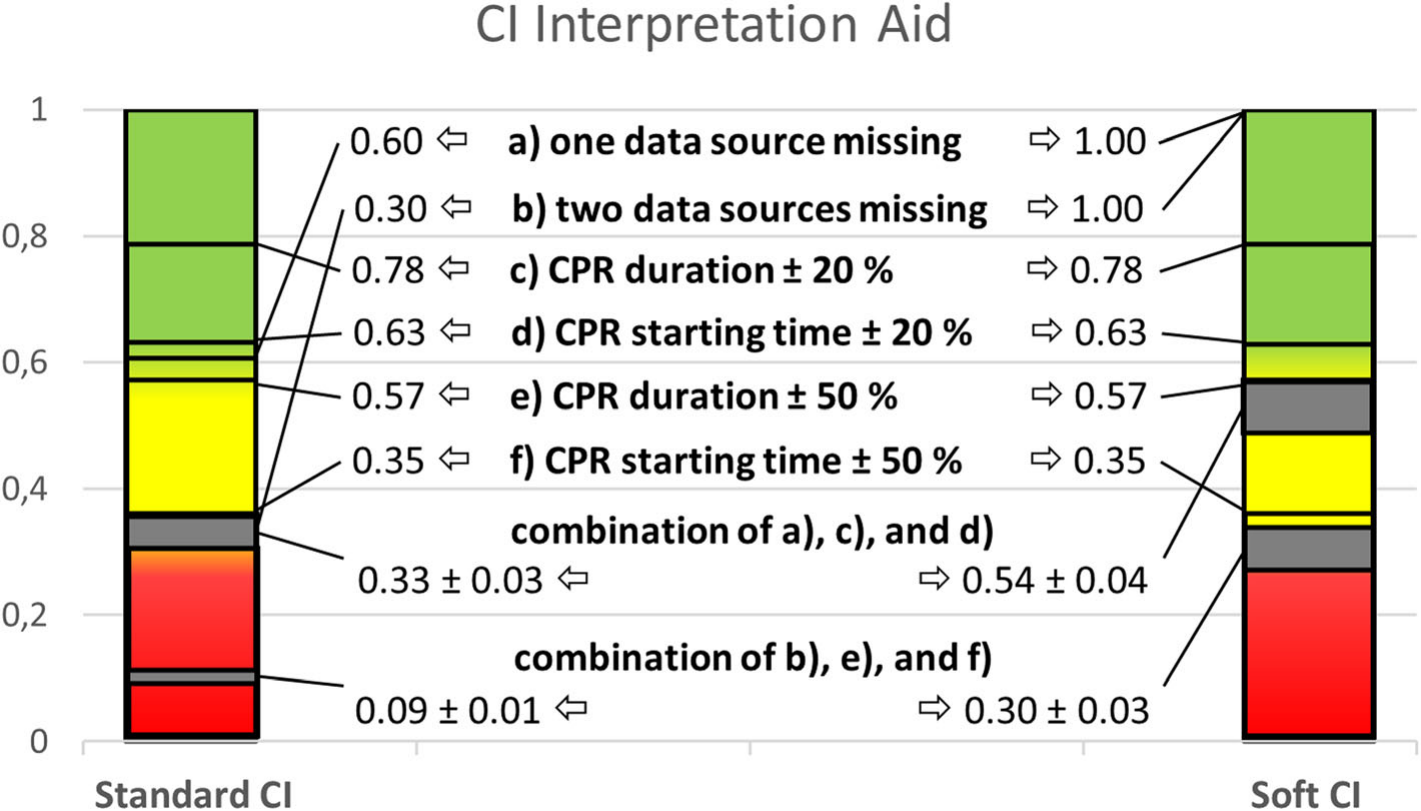
Interpretation aid for the CI. Graphical display of values for the standard CI (left column) and soft CI (right column) originated from empty data sources (one or two), varying CPR duration or starting time (± 20% and ± 50% relative to the remaining data sources, respectively), and a combination thereof. Column colors code for the overall CI rating according to the opinion of the authors: CI values of 1 to 0.6 are deemed to represent good documentation reliability (green area), values between 0.3 and 0.6 are intermediate (yellow area), and values below 0.3 represent poor documentation quality (red area)

To assist in the interpretation of standard / soft CI findings, typical documentation aberrations (missing data, differences in CPR starting time, and / or duration) and the resulting CI values are displayed in Table 3 and Fig. 2).

**Table 3:** Reductions of standard and soft CI as a function of missing or varying information on CPR starting time or duration, or a combination of these characteristics, respectively

	Standard CI is reduced to …	Soft CI is reduced to …
CPR not represented in …		
(1) one data source	0.6	1.0
(2) two data sources	0.3	1.0
Starting time varying by …		
(3) ± 20%	0.629	0.629
(4) ±50%	0.35	0.35
CPR duration varying by …		
(5) ± 20%	0.783	0.783
(6) ± 50%	0.567	0.567
Combination of (1), (3), and (5)	0.3–0.35	0.5–0.583
Combination of (2), (4), and (6)	0.080–0.100	0.267–0.333

### Daytime effect

To assess whether documentation quality would vary between day and night shifts, both the standard and soft CI were compared between CPR incidents during daytime (6:00 AM until 09:59 PM) to those during nighttime (10:00 PM until 5:59 AM) in cases with known CPR starting times.

### Latency of documentation

The PDMS allowed further to trace the latency between the initiation of CPR and the time point of entering the incident into the PDMS. Thus, whenever a time point of CPR initiation could be found in a given data source, the time interval until its documentation was noted. In a second step, this time latency was correlated to data quality, namely the soft CI.

### Cardiac rhythm and electrical defibrillation

During CPR episodes, in the physicians’ log, the nurses’ log, and the vital signs and therapy record, entries of cardiac rhythm and electrical defibrillations were reviewed and correlated.

### Statistical analysis

Descriptive data analysis was performed on Microsoft Excel 2013 (Microsoft Corp., Redmond, WA). For time related data and CI, median values, interquartile ranges (IQR), 10th and 90th percentiles are provided.The comparative analysis was performed using SPSS Statistics 26 (IBM, Armonk, NY). For the comparison between day and night shifts, the Mann-Whitney test was applied. The correlation of documentations’ time latency to the CI, the Pearson and Spearman correlation analysis was used. Differences were considered to be statistically significant, if P was lower than 0.05.

## Results

### Case numbers

The search for CPR patients revealed 245 hits. Twenty-two cases were removed from the study since the CPR mentioning had been deleted after false documentation (which was still visible due to the traceability of all changes within the electronic record). In addition, 42 cases have been omitted because the resuscitation was partly performed outside the ICU. Sixteen cases were removed due to the resuscitation being solely pharmacologic without obvious need for chest compressions. Thus, 165 cases were included into the study.

**Fig. 3:**
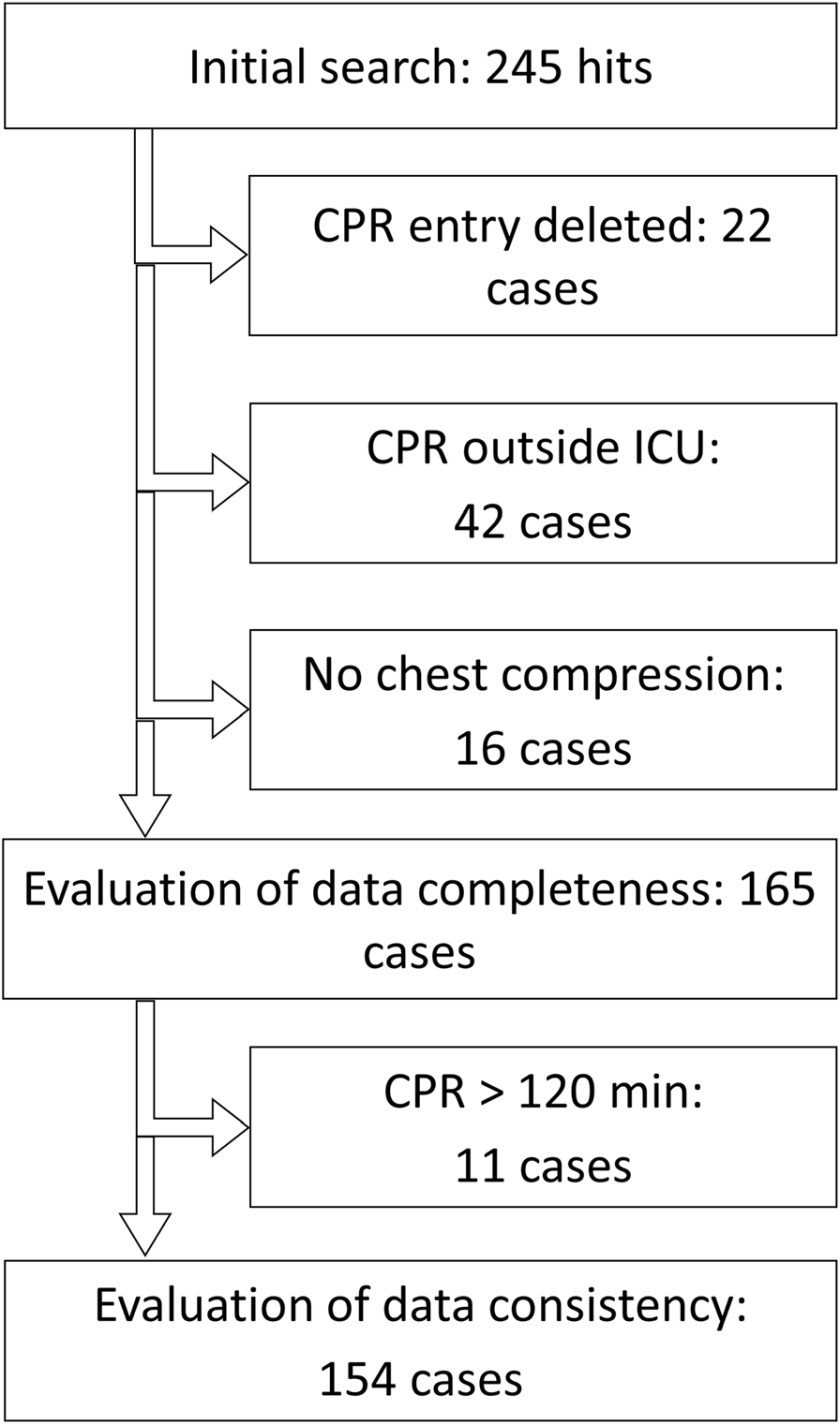
Case number inclusion and exclusion

For analysis involving the CI, CPR procedures with a documented duration of more than 120 min were disregarded, what was the case in 11 incidents. Therefore, 154 cases could be included into the evaluation of the CI (Fig. 3).

### Data completeness

#### Frequency of documentation in data sources

Out of the 165 CPR episodes included into the study, 33 (20%) were represented in all four examined data sources. Seventy-four (45%) cases were reported in three sources, 27 (16%) in two, and 16 (10%) in only one data source. There were 15 CPR incidents (9%) for which no data in any of the analyzed documentation locations could be found.

Incident documentation was most likely to be found in the physician’s log (76%), followed by the nurse’s log (69%) and the CPR incident form (60%). From vital signs and therapeutic measures, 55% of CPR incidents could be read out.

#### Completeness of time related documentation

In most cases, time related data on CPR incidents were found within at least one of the evaluated data sources. However, no information could be found in 23% of cases concerning CPR duration and 13% concerning CPR starting time. In 9%, neither data on duration nor starting time could be retrieved (Table 4).

**Table 4:** Availability of data on CPR duration and / or starting time in at least one of the four data sources

	Starting time available	Starting time not available	Sum
CPR duration available	120 (73%)	7 (4%)	127 (77%)
CPR duration not available	23 (14%)	15 (9%)	38 (23%)
Sum	143 (87%)	22 (13%)	165 (100%)

To conclude on time-related CPR documentation reliability, data need to be available from at least two data sources within individual incidents. This was applicable in 90 cases (54%) for the starting time and in 74 (45%) for CPR duration.

### Data consistency

#### Differences in time related documentation

##### Starting time

For those 90 CPR incidents, in which a comparison of different specifications of the starting time was possible, the median difference between the earliest and the latest starting time for individual cases was 10 min with an IQR of 18.5, a 10th percentile of 0.0 and a 90th percentile of 49.5 min.

##### CPR duration

The CPR duration differed by a median value of 5 min (IQR 17.3, 10th percentile 0.0, 90th percentile 124.0 min) between the shortest and the longest interval mentioned for individual cases.

##### Time latency of documentation

Of the 154 cases included into data consistency analysis, 16 did not contain any information on CPR initiation, and thus could not be included into the documentation latency evaluation. For the remaining 138 cases, the median latency was 125.5 min (IQR 188.3, 10th percentile 17.0, 90th percentile 382.3 min).

#### Consentaneity index

##### Standard consentaneity index

**Fig. 4:**
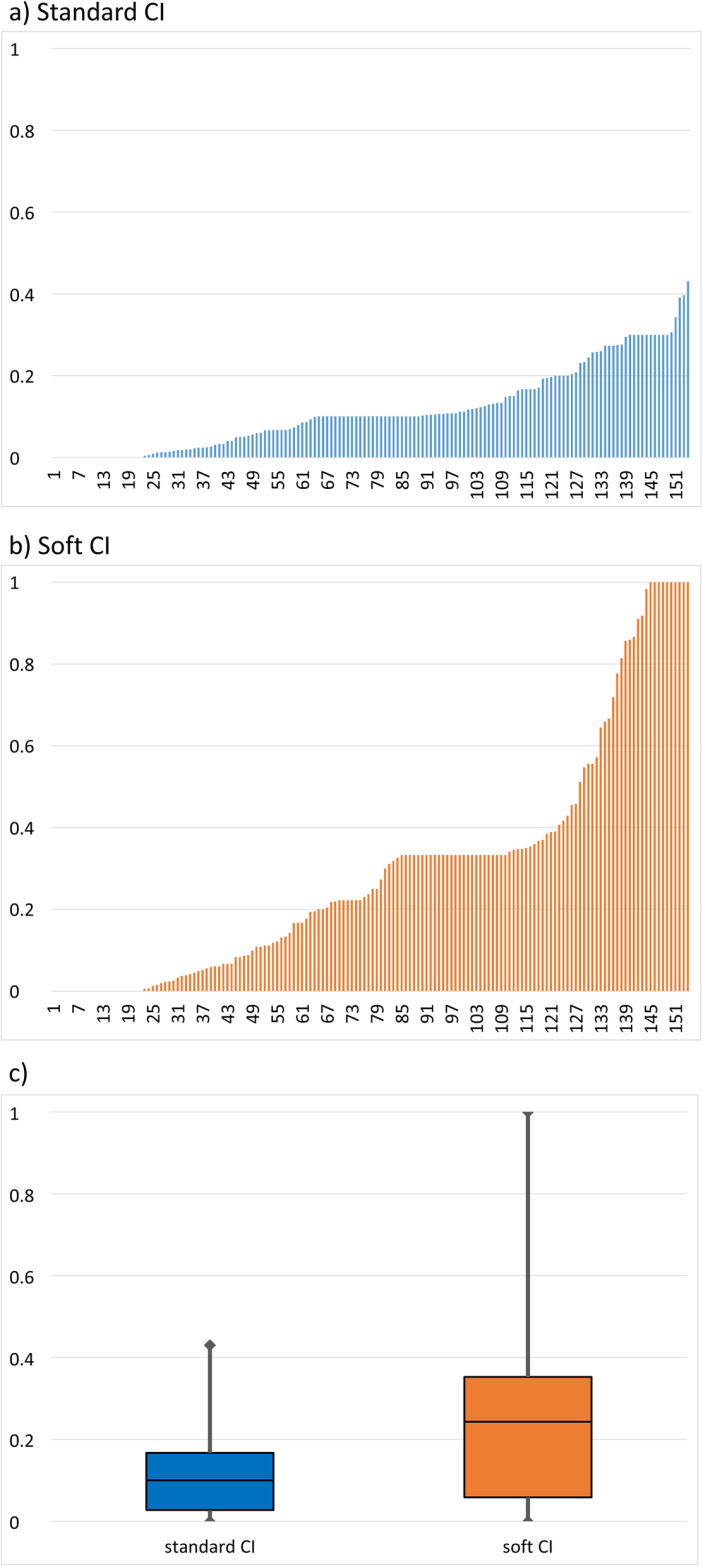
Results for standard Consentaneity Index (CI). **a**, **b** Bar graph of standard CI (**a**) and soft CI (**b**) results by individual cases, sorted by increasing CI. **c** Box plot of standard and soft CI (median, quartiles and range)

The standard CI could be evaluated in 154 cases. The actual CI ranged from 0 to 0.431. The median CI was 0.100 (IQR 0.140, 10th percentile 0.000, 90th percentile 0.298) (Fig. 4a and c).

##### Soft CI

The soft CI focusses on the consistency of available data and is not influenced by empty data sources for a given CPR episode. Therefore, the results for the soft CI are higher: The median soft CI was found to be 0.244 (IQR 0.298, 10th percentile 0.000, 90th percentile 0.859), with a data range of 0 to 1 (which was reached in 10 cases) (Fig. 4b and c).

##### CI in day and night shift

Within the 132 cases with known CPR starting time, there were no differences between CPRs during day (*n* = 88) and night shifts (*n* = 44) regarding the median standard CI (day 0.100 (IQR 0.097) vs. night 0.107 (IQR 0.166)) or the soft CI (0.333 (IQR 0.246) vs. 0.330 (IQR 500)) (*P* = 0.48 and *P* = 0.75, respectively, Mann-Whitney-Test).

#### Consistency of cardiac rhythm and defibrillation data

The initial cardiac rhythm was available from the physicians’ log in 47.9%, from the nurses’ log in 29.7%, and from the incident reporting form in 9.7%. In 68 cases (41.2%), the cardiac rhythm was absent in all data sources, and in eight cases (4.8%) the different data sources for individual cases contained conflicting information. Defibrillation was performed in 33 cases (20.0%). A shockable rhythm was mentioned in 26 cases (15.7%), which was treated by defibrillation in 21 cases. Of the remaining 139 cases without explicit mentioning of a shockable rhythm, in twelve cases defibrillation was documented (four of those in cases with documentation of a non-shockable rhythm and eight with no rhythm available). Thus, according to the PDMS record, the rate of correct defibrillation or correct refraining from defibrillation was 89.7% (148 cases). In 17 cases (10.3%), according to the documentation, shockable cardiac rhythms were not associated with defibrillator use and vice versa (Table 5).

**Table 5:** Cardiac rhythm and electrical defibrillation. * Four cases with documentation of non-shockable rhythm, eight cases with missing data. VF = cardiac fibrillation, VT = ventricular tachycardia

	VF / VT	No VF / VT	Sum
Defibrillation	21 (12.7%)	12 (7.3%) *	33 (20%)
No Defibrillation	5 (3.0%)	127 (77.0%)	132 (80%)
Sum	26 (15.7%)	139 (84.3%)	165 (100%)

### Correlation between documentation latency and data quality

**Fig. 5:**
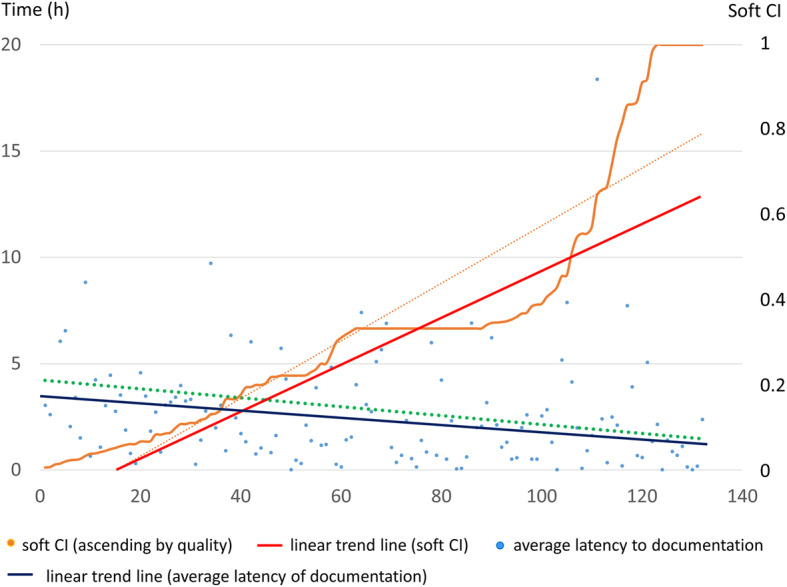
Correlation between soft CI and latency to documentation

A short time interval between CPR initiation and documenting the incident was positively correlated to a higher soft CI. The Pearson analysis resulted in *r* = − 0.16 (*P* = 0.67), the Spearman analysis showed *r* = − 0.268 (*P* = 0.002) (Fig. 5).

## Discussion

### Importance of reliable CPR documentation

Much of our current knowledge on CPR originates from large scientific data registries. To date, the time-consuming manual data entry into these repositories is a drawback in many of these systems. In operating rooms, intensive care as well as out-of-hospital emergency settings, automated collection of patient vital parameters, combined with semi-automated or manual documentation of therapeutic measures and further details, in electronic PDMS is on the rise. If such a PDMS is in place, the relevant data that are needed for research purposes are readily available in electronic form. Automatic data transfer from PDMS to scientific registries, however, lacks the plausibility check that is associated with manual data handling, thus bearing the risk of poor data quality. In a nutshell, data quality of electronic routine medical data collection is paramount for its use for scientific purposes.

In this study, the authors evaluated the reliability of CPR related documentation within a PDMS in three independent ICUs of a single university hospital. Special interest was put on the time of CPR onset and its duration, two relevant parameters for quality assurance and research. Both data comprehensiveness and data consistency showed shortcomings. For the quantification concerning the reliability of PDMS entries, a numeric measure, the Consentaneity Index (CI), was introduced.

As demonstrated in this work, in many cases the medical record provided conflicting information on the starting time of CPR and its duration. Thus, the exact CPR interval could not be reliably determined. This renders it difficult or even impossible, to correlate other clinical findings or therapeutic interventions to the CPR intervals for scientific purposes. Further, it seems advisable not to relay on a single data source when the CPR interval is intended to be read from the PDMS documentation (e.g., when exporting PDMS data to scientific registries). Whenever intervals of running CPR are to be identified from routine medical records, the authors propose to choose time intervals with a soft CI of 1, i.e., all CPR mentioning data sources are in positive accordance.

Another purpose for data use relying on accurate time-related CPR documentation is process quality management. For example, in-hospital VF should be defibrillated within less than 3 min [16]. Therefore, the latency between circulatory arrest and the first defibrillation is part of the Utstein template for CPR documentation. The Utstein authors rate the importance of this parameter as follows: “In the hospital, the time interval from collapse/arrest to first defibrillation attempt may be the most important process indicator of effective response when VF is the initial cardiac arrest rhythm” [17]. If, as found in this study, individual data on CPR starting time differs in every second case by more than 10 min, its use for benchmarking the three-minute-defibrillation requirement seems questionable.

### CPR documentation quality in the literature

In the literature, there are numerous studies dealing with the quality of CPR documentation in different settings. Most of these studies focus on the completeness of CPR data in the medical record [3, 4, 6–9]. A few studies address data consistency: Frisch et al. evaluated differences in time discrepancies between the medical record and time-stamped audio recordings in out-of-hospital cardiac arrest situations [1]. A combined registry analysis and single center chart review revealed implausible (negative or zero) resuscitation time intervals in in-hospital cardiac arrest [2]. Two studies suggest, the CPR documentation quality can be ameliorated: an intervention study involving a CPR training session showed, that both CPR documentation as well as compliance with CPR guidelines improved [8]. In a pediatric setting, the introduction of weekly resuscitation case discussions did improve both CPR guideline coherence and CPR documentation [9]. However, none of these studies is dealing explicitly with data quality of CPR recordings in the ICU setting.

The PDMS used in this study allows the entry of CPR related data in several data forms. While these options are facilitating daily recording, it allows the medical record to be fragmentary and even contradictory.

### Data completeness

Despite the fact that an up-to-date PDMS was in use on the three ICUs in this study, there were lacks in data completeness. In 18% of reviewed cases, either CPR starting time or duration were not available from the electronic record (Table 4). Only in a minority of cases (20%), information on the individual CPR could be found in all four examined data sources.

### Data consistency

The analysis of data consistency showed contradictions within individual cases concerning the starting point and the CPR duration. In addition, there were some cases with inconsistencies between the indication for electrical defibrillation, as read from the heart rhythm documentation, and the documented use of the defibrillator.

Data consistency was more favorable if the CPR documentation was completed shortly after the event. Longer latencies between resuscitation and data entry were associated with lower values of the soft CI.

### Improving CPR documentation

The results of this work suggest that electronic CPR documentation in the ICU setting needs improvement. This purpose could be addressed by technical and non-technical measures. From a technical point of view, the user friendliness of CPR documentation can be improved by designing smart CPR event forms for instant (ideally real-time) use. An integration of those event forms in the physicians’ and nurses’ log could avoid data gaps and contradictions. Non-technical approaches consist particularly of the attempt to strengthen the importance of documentation in CPR training and team organization. Anecdotal experience suggest that CPR documentation works best, if one team member is taking over this task without other responsibilities during the CPR process. However, fulfilling medical necessities remains the number one priority during resuscitation efforts, and documentation must be subordinated, if human resources are limited. The CI could be a valuable tool to monitor the effect of any improvement efforts concerning CPR documentation in the ICU.

### Limitations

There are some limitations to our study. It was not possible to correlate any documentation to the factual proceedings of the CPR situations. Thus, no conclusion of the validity of the PDMS content can be made.

Some CPR events will have been missed by the study due to the search strategy applied. If the respective TISS documentation of the code was absent, it was not included into the evaluation. Since there is no reason to assume that those missing cases should differ from the ones included into the study, a selection bias seems unlikely.

Under certain constellations with implausibly long CPR episodes (e.g., according to the CPR incident form), the soft CI can take false high values. Therefore, CPR documentations lasting longer than two hours have been excluded from the analysis.

## Conclusions

The assumption that the use of an electronic PDMS would automatically result in a reliable and unambiguous documentation of CPR attempts is false. Real-life data in this study show relevant proportions of missing and inconsistent data in the ICU setting. Thus, documentation of CPR events needs further attention. The Consentaneity Index is suggested as a tool for documentation quality analysis and monitoring of improvements.

## Data Availability

The datasets used and/or analyzed during the current study are available from the corresponding author on reasonable request.

## References

[1] Frisch A, Reynolds JC, Condle J, Gruen D, Callaway CW (2014). Documentation discrepancies of time-dependent critical events in out of hospital cardiac arrest. Resuscitation.

[2] Kaye W, Mancini ME, Truitt TL (2005). When minutes count--the fallacy of accurate time documentation during in-hospital resuscitation. Resuscitation.

[3] Sundermann ML, Salcido DD, Koller AC, Menegazzi JJ (2015). Inaccuracy of patient care reports for identification of critical resuscitation events during out-of-hospital cardiac arrest. Am J Emerg Med.

[4] Sukul D, Kamphuis LA, Iwashyna TJ, Bradley SM, Chan PS, Sinha SS, Nallamothu BK (2017). Clinical documentation of in-hospital cardiac arrest in a large national health system. Resuscitation.

[5] Stewart JA (2016). Problems with time-interval data from in-hospital resuscitation records. Int J Cardiol.

[6] Lam S, Marcos K, Thangavelu M, Doerr D, Bhalala U (2018). 347: quality of critical event documentation in a Children's hospital. Crit Care Med.

[7] Bakhsh AA, Bakhsh AR, Karamelahi ZA, Bakhsh AA, Alzahrani AM, Alsharif LM, Sharton YM, Alotaibi AK, Basharahil KO (2018). Communicating resuscitation. The importance of documentation in cardiac arrest. Saudi Med J.

[8] Nevrekar V, Panda PK, Wig N, Pandey RM, Agarwal P, Biswas A (2017). An interventional quality improvement study to assess the compliance to cardiopulmonary resuscitation documentation in an Indian teaching hospital. Indian J Crit Care Med.

[9] Root L, van Zanten HA, den Boer MC, Foglia EE, Witlox RSGM, Te Pas AB (2019). Improving guideline compliance and documentation through auditing neonatal resuscitation. Front Pediatr.

[10] Grigg E, Palmer A, Grigg J, Oppenheimer P, Wu T, Roesler A, Nair B, Ross B (2014). Randomised trial comparing the recording ability of a novel, electronic emergency documentation system with the AHA paper cardiac arrest record. Emerg Med J.

[11] Grundgeiger T, Albert M, Reinhardt D, Happel O, Steinisch A, Wurmb T (2016). Real-time tablet-based resuscitation documentation by the team leader. Scand J Trauma Resusc Emerg Med.

[12] Peace JM, Yuen TC, Borak MH, Edelson DP (2014). Tablet-based cardiac arrest documentation. Resuscitation.

[13] Stewart JA, Short FA (1999). Time accuracy of a barcode system for recording resuscitation events. Resuscitation.

[14] Reis Miranda D, Schaufeli W, Rijk A de (1996). Simplified therapeutic intervention scoring system: the TISS-28 items--results from a multicenter study. Crit Care Med.

[15] Burchardi H, Specht M, Braun J, Schleppers A, Martin J (2004). OPS-Code 8–980 “Intensivmedizinische Komplexbehandlung”: Stellungnahme, Inhalte und Kodiervorschriften.

[16] Soar J, Böttiger BW, Carli P, Couper K, Deakin CD, Djärv T, Lott C, Olasveengen T, Paal P, Pellis T, Perkins GD, Sandroni C, Nolan JP (2021). European Resuscitation Council Guidelines 2021: Adult advanced life support. Resuscitation.

[17] Jacobs I, Nadkarni V, Bahr J, Berg RA, Billi JE, Bossaert L, Cassan P, Coovadia A, D'Este K, Finn J, Halperin H, Handley A, Herlitz J, Hickey R, Idris A, Kloeck W, Larkin GL, Mancini ME, Mason P, Mears G, Monsieurs K, Montgomery W, Morley P, Nichol G, Nolan J, Okada K, Perlman J, Shuster M, Steen PA, Sterz F, Tibballs J, Timerman S, Truitt T, Zideman D (2004). Cardiac arrest and cardiopulmonary resuscitation outcome reports. Circulation.

